# Did Dental Insurance Expansion Improve Dental Care Needs Among Korean Adults? Difference in Difference Analysis

**DOI:** 10.2188/jea.JE20200596

**Published:** 2023-02-05

**Authors:** Nam-Hee Kim, Hawazin W. Elani, Ichiro Kawachi

**Affiliations:** 1 Department of Dental Hygiene, Wonju College of Medicine, Yonsei University, Wonju, Republic of Korea; 2 Department of Social and Behavioral Sciences, Harvard T.H. Chan School of Public Health, Boston, MA, USA; 3 Department of Dental Hygiene, College of Software and Digital Healthcare Convergence, Yonsei University, Wonju, Republic of Korea; 4 Department of Oral Health Policy and Epidemiology, Harvard School of Dental Medicine, Boston, MA, USA

**Keywords:** insurance, unmet dental needs, older adults, dentures, difference-in-difference, triple-difference, quasi-experimental

## Abstract

**Background:**

In 2012, the Korean government expanded dental insurance for the elderly to promote improved access to dental care. We examined the causal effect of this policy on dental care needs, focusing on low-income older adults.

**Methods:**

We compared data before and after policy implementation using double difference (DD) and triple difference (DDD) analyses. We used the nationally representative data from the Korea National Health and Nutrition Examination Survey from 2010 and 2016–2018. Individuals aged ≥65 years were included in the treatment group, and individuals aged <65 years were included in the control group.

**Results:**

Dental insurance expansion was associated with a paradoxical increase in perceived unmet dental needs among elderly individuals (8.8 percentage points increase, 95% CI: 4.7 to 13.0). However, there were improvements in dental prosthetics outcomes (denture wearing [4.0 percentage points, 95% CI: 0.2 to 7.9] and dental implants [5.0 percentage points, 95% CI: 2.1 to 7.9]; *P* < 0.01). Upon analyzing low-income elderly individuals using DDD analysis, we found that the insurance expansion led to a 21.6% smaller increase in unmet dental needs among low-income adults, compared to high-income adults (95% CI, −35.0 to −8.5; *P* < 0.01).

**Conclusion:**

Dental insurance expansion in South Korea resulted in improvements in access to dental prosthetic services overall. It also led to a smaller increase in unmet dental needs among low-income older adults, compared to high-income adults.

## INTRODUCTION

Out-of-pocket (OOP) expenses create financial barriers to access to dental health care, especially for socioeconomically disadvantaged populations, resulting in neglected oral health.^1^^,^^2^ Older individuals in South Korea are especially at risk of poverty due to inadequate social security,^3^ making them vulnerable to chronic poor oral health, which in turn affects their quality of life.^4^^,^^5^ The prevalence of unmet dental needs is distinctly high among low-income adults, elderly individuals, immigrants, and individuals with disabilities.^6^^–^^12^ Additionally, limited dental resources are associated with high unmet dental needs at the community level.^6^^,^^13^^,^^14^ Insurance for dental care is expected to improve oral health outcomes and shrink income-based oral health inequalities.

There is growing consensus that expanded dental insurance coverage not only increases dental care utilization but also improves oral health outcomes. There is growing evidence from Medicaid expansion under the Affordable Care Act (“Obamacare”) in the United States that insurance coverage improves access to dental care among low-income adults.^15^^–^^17^ In terms of emergency dental (ED) visits, Medicaid expansion led to increase in ED utilization for dental care for non-elderly low-income population. However, in states that expanded Medicaid and provide adult dental benefits dental ED visits declined (by 14 percentage points).^18^ The expansion of Medicaid coverage also reduced unmet dental care needs by 13 percentage points among low-income adults.^19^ Furthermore, Medicaid beneficiaries with dental coverage were 9.5 percentage points less likely to have untreated caries.^20^

However, despite improved dental insurance coverage, an “inverse care law” in dental care could stubbornly persists.^21^^–^^23^ Such a trend has been observed in Canada, where individuals with the highest need for medical care end up accessing the required services the least, while those with the lowest need for dental care use the required services the most.^22^ However, contrasting findings have also been reported, indicating no inverse dental care law for adults receiving National Health Service dental care in the United Kingdom.^21^ In Canada, the lack of universal dental health care services may explain the finding of an inverse care law there.

South Korea has been providing universal health coverage for over 30 years; however, dental service coverage is limited. National health insurance with dental coverage includes treatment for dental caries and periodontal disease with about 30–50% OOP expense and limited services. However, dental prosthetic treatments were not covered by insurance and required OOP expenses. In 2007–2009, approximately 36% of older adults had unmet dental needs due to the OOP costs associated with dental care.^24^ In 2012, the Korean government expanded dental insurance for elderly individuals to promote improved access to dental care. This National Health Insurance scheme covered dental prosthesis including partial or full dentures and dental implants. It also included 50–70% reduction in OOP for all individuals aged ≥65 years, irrespective of the socioeconomic status.

In previous research, we reported that expansion of dental insurance in Korea resulted in improvements in income-based inequalities (on the absolute scale) in self-reported oral health, but persistence of unmet dental needs among older adults. In addition, inequalities on the relative scale persisted for self-reported oral health and actually widened for unmet dental needs over time.^25^^,^^26^ Hence, dental insurance expansion in Korea produced some mixed effects for oral health inequalities. One reason is that despite the expansion in access to care, low income older adults may not have been able to utilize dental care due to other persisting barriers, such as high costs of certain procedures (especially prosthetic dentistry) and low oral health literacy.^27^^,^^28^ In the present study we sought to extend our previous research by considering the heterogeneity in the effects of the policy according to income level, using a triple-difference design.

To identify the effect of expanded dental insurance benefits for elderly individuals, we assumed that there would be no differences between the eligible and non-eligible groups before and after the introduction of the dental benefits policy in 2012. In addition, we assumed that there would be no difference between the low- and high-income eligible groups before and after the policy. We also assumed that other predictors of oral health outcomes do not change among the non-eligible groups and that differences between the observed and expected outcomes were attributed to the effects of coverage. Such quasi-experimental approaches have a common identification strategy that use differences in oral health among groups before coverage changes to predict subsequent differences in oral health that would be expected with no changes in coverage.^29^ In the present study, we sought to examine the causal effect of dental insurance expansion on dental care needs for the elderly, especially focusing on older, low-income adults.

## METHODS

### Study design and data source

We conducted a quasi-experimental analyses using double difference (DD) and triple difference (DDD) to identify the causal effects of a policy change. Nationally representative repeated cross-sectional data from the Korea National Health and Nutrition Examination Survey (KNHANES), conducted by the Korea Centers for Disease Control and Prevention (KCDC), were analysed. Multi-stage clustered probability sampling was used to collect data. Data from 2016–2018 was considered as a single point data. The primary sampling units (PSUs) were drawn from approximately 200,000 geographically defined PSUs including urban and rural areas of the country. These units consisted of an average of 60 households, and 20 final target households were sampled for each PSU using systematic sampling.^30^

A difference-in-difference analysis (ie, DD analysis) was performed to compare the effect of dental insurance expansion for elderly individuals. We used data from before (2010) and after (2012, 2014, 2015, and 2016–2018) policy implementation. Our study population was restricted to adults aged 50–80 years to ensure an age range for comparison. Our treatment group included individuals aged ≥65 years (aged 65–80 years) who were eligible for the elderly benefits (before, *n* = 1,478; after, *n* = 8,136). The control group included individuals aged <65 years (aged 50–64 years) who were not eligible for the benefits (before, *n* = 1,795; after, *n* = 8,834).

Triple difference (DDD) analysis was performed to assess the effect of expansion in the low-income group. The treatment group comprised eligible individuals aged ≥65 years with low incomes (before, *n* = 1,064; after, *n* = 5,118). The control group for the DDD analysis included individuals aged ≥65 years with high incomes who were eligible for the elderly dental insurance benefits (before, *n* = 699; after, *n* = 5,118).

### Study variables

The primary outcome was a measure of unmet dental needs, assessed using face-to-face interviews with ‘yes or no’ response options to the following question: ‘Did you need dental care but were unable to receive dental treatment within the past year?’. Variables linked to access to dental care and oral health outcomes related to unmet dental needs were included as secondary outcomes. Access to dental care included all three levels of prevention: primary prevention (oral examination, dental scaling, and/or fluoride application) and secondary and/or tertiary prevention (periodontal treatment and tooth extraction). Oral health outcomes included clinical oral health outcomes (periodontitis, denture wearing, denture needs, dental implant, number of remaining teeth, and edentulism) and perceived oral health outcomes related to quality of life (self-reported poor oral health). Perceived unmet dental needs has been validated in terms of clinically determined dental needs.^31^

All variables for access to dental care were assessed based on whether individuals had availed any dental care in the last 1 year. Oral health outcomes were clinically evaluated by trained public health dentists in accordance with World Health Organization guidelines.^32^ Periodontitis included community periodontal index (CPI) score 3 and/or 4; denture wearing status was based on whether the individual had one or more partial and/or complete dentures in the upper and/or lower arch; and need for dentures was based on whether patients had a visible requirement for one or more partial and/or complete dentures in any dental arch. Dental implant status recorded if the individual had one or more dental implants in the upper and/or lower arches. The total number of remaining teeth was counted excluding wisdom teeth. Perceived oral health outcome was assessed by recording perceived status of oral health problems (very good, good, moderate, bad, and very bad).

We adjusted for sex, educational attainment (elementary school, middle school, high school, or college or over), and income level (low, low-middle, middle-high, or high). Income was equivalized for household size and grouped into four categories by quartiles provided by the KCDC^30^—lowest 25% (low-income), 25–50% (middle-low), 50–75% (middle-high) and upper 25% (high-income).

### Statistical analysis

**Figure 1.**
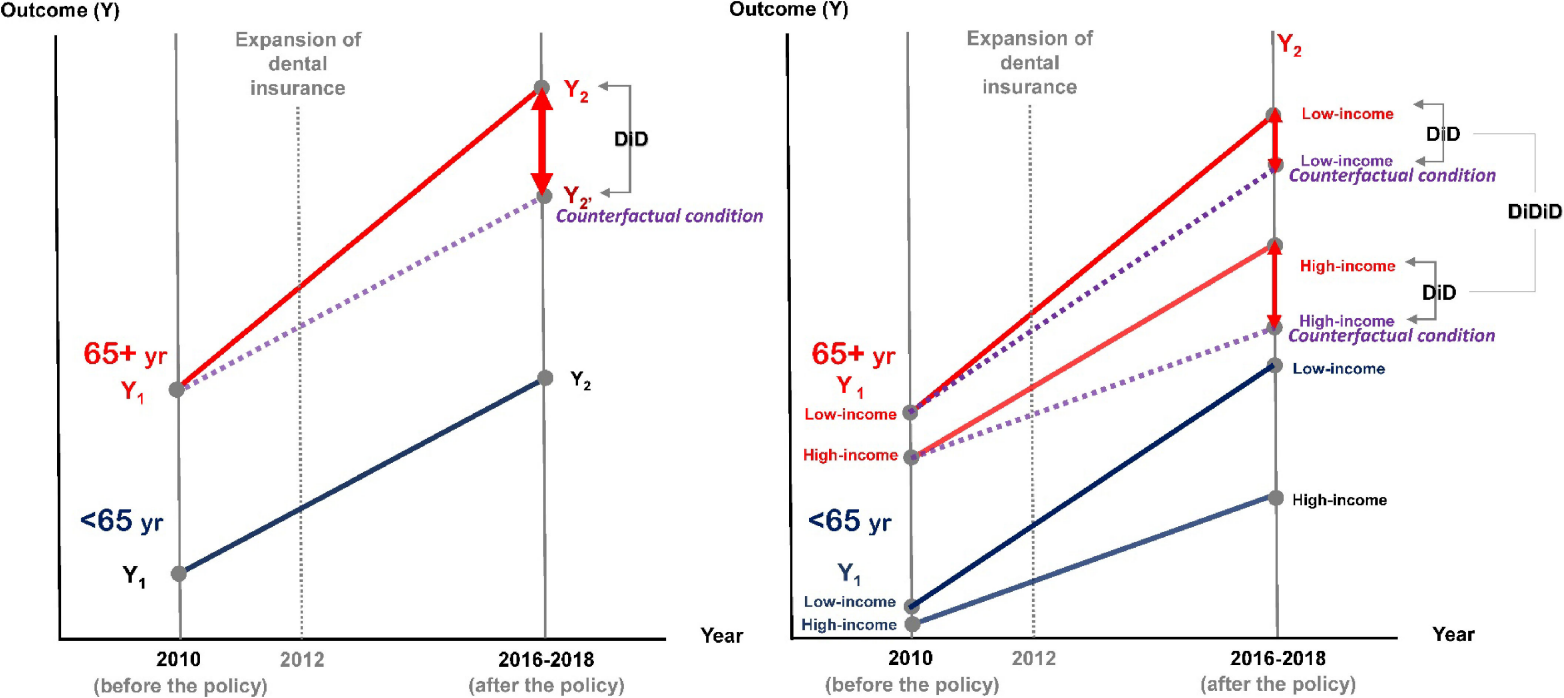
Diagram of DD and DDD analyses. Solid red line: eligible group (65+ yr); Solid blue line: non-eligible group (<65 yr); Dotted purple line: counterfactual condition

Oral health outcome descriptive statistics were calculated before and after policy implementation for individuals aged ≥65 and <65 years. We performed DD analysis by estimating the DD coefficient using a standard regression model^33^:

Yi=β0+β1×eligiblei+β2×year+β3×(eligiblei×year)

where for individual Y is the oral health outcome of interest (eg, Y = 1 if the person reported unmet dental needs), ‘eligible (treatment)’ is a dummy variable for whether the person was eligible for elderly dental insurance benefits (1 = eligible), and ‘year’ pertains to the policy period (pre-policy = [2010]; post-policy = [2012, 2014, 2014, 2015, and 2016–2018]). The causal parameter of interest is β3, which is equivalent to the quantity [(Ytreat_2_ − Ytreat_1_) − (Ycont_2_ − Ycont_1_)] or how much more unmet dental needs rates changed in the treatment group relative to the control group (Figure 1). Further, robust standard errors were considered, and all regression models were adjusted for sex, education attainment, and income level.

Additionally, we calculated the prevalence rates for outcomes among low-income elderly individuals and high-income elderly individuals, both before and after the policy was enacted. These rates were further analyzed in DDD analysis, using linear regression and the interaction term (treatment * year * income). The DDD equation was:

$$\begin{array}{rl} \text{Y}i & =\beta 0+\beta 1\times \text{eligiblei}+\beta 2\times \text{incomei}\\ & \;+\beta 3\times (\text{eligiblei}\times \text{incomei})+\beta 4\times \text{year}\\ & \;+\beta 5\times (\text{year}\times \text{eligiblei})+\beta 6\times (\text{year}\times \text{incomei})\\ & \;+\beta 7\times (\text{year}\times \text{eligiblei}\times \text{incomei}) \end{array}$$

where β7, the key parameter of interest, represents the causal effect of the policy on oral health outcomes for the low-income group compared to the high-income group (Figure 1).

Finally, several sensitivity analyses were also performed to check the robustness of our results. These included placebo intervention period using data from 2007–2010, alternative control group including individuals aged 34–49 years, and a placebo outcome using perceived obesity. Perceived obesity was assessed using self-reported responses to the following question: ‘How would you describe your body shape?’ Individual responses of ‘very thin’, ‘thin’, and ‘normal’ were categorized as ‘not obese’ and assigned a value of 0; whereas responses of ‘slightly obese’ and ‘very obese’ were categorized as ‘obese’ and assigned a value of 1.^34^

We used the KNHANES survey weight to account for the complex survey design. We used STATA statistical software (Release 15; STATA Corp., College Station, TX, USA) for all statistical analyses.

### Ethics approval and consent to participate

This study used publicly available data from the KNHANES conducted by the KCDC. All participants of the KNHANES agreed to participate in the survey. The KNHANES was approved by the Institutional Review Board (IRB) of the KCDC. This study was deemed to be “not human subjects research” because it used anonymous, public-use secondary data, so it was exempt from IRB approval.

## RESULTS

### Distribution of the study population and prevalence rates of outcomes

Table 1 summarizes the distribution of key variables in the treatment and control groups before and after the introduction of dental insurance expansion policy, indicating that the treatment group (individuals eligible for expanded access to dental services) had lower incomes and education status (*P* < 0.01) (eTable 1).

**Table 1.** Distribution of the study population (%)

	Before (2010 year)				After (2012–2018 year)			
	
	total	Control^a^ (<65)	Treated^b^ (≥65)	*P*	total	Control^a^ (<65)	Treated^b^ (≥65)	*P*
Number of participants	3,273	1,795	1,478		16,997	8,834	8,136	
**Age**, mean (SE)		56.7 (0.10)	72.1 (0.12)			56.9 (0.05)	72.8 (0.06)	
**Sex**								
Men	1,427	43.3	43.9	0.74	7,309	42.9	43.1	0.86
Women	1,846	56.7	56.1		9,688	57.1	56.9	
**Education**								
Elementary	1,535	35.1	68.5	<0.01	6,341	24.1	60.7	<0.01
Middle school	544	22.5	12.0		2,548	19.1	14.0	
High school	673	29.4	12.9		3,906	33.9	16.3	
College over	312	13.1	6.6		2,500	22.9	9.0	
**Income**								
Low^c^	1,064	16.4	53.3	<0.01	5,118	13.8	48.4	<0.01
Middle-low	798	26.4	22.9		4,446	25.7	27.2	
Middle-high	658	26.7	12.9		3,589	27.3	14.9	
High^d^	699	30.5	11.0		3,688	33.3	9.6	

Unweighted number of the sample and weighted %.

^a^Control group (<65) included individuals aged 50–64 years.

^b^Treatment group (≥65) included individuals aged 65–80 years.

^c^Treatment group (before and after) for DDD analysis.

^d^Control group (before and after) for DDD analysis.

**Figure 2.**
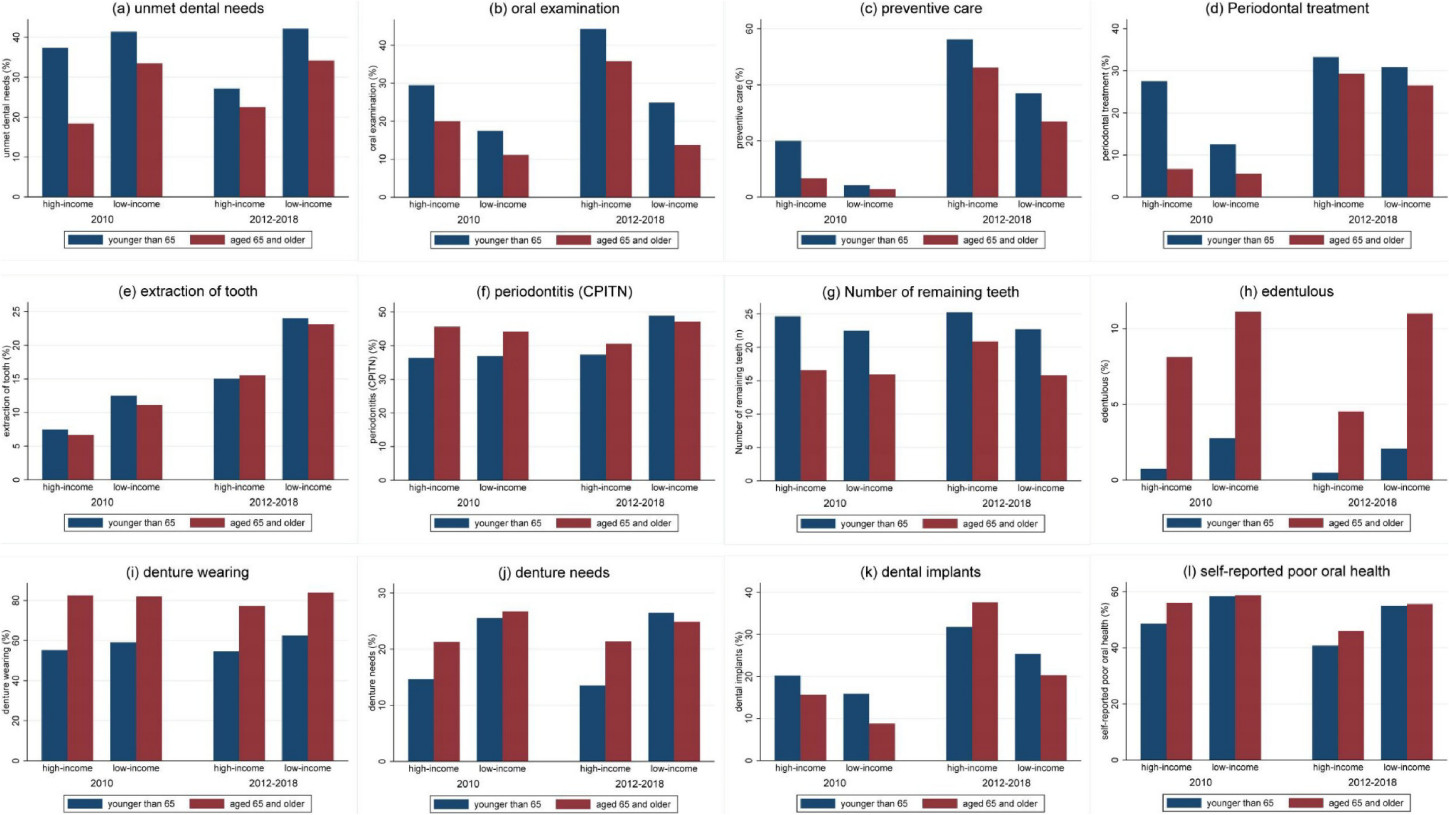
Unadjusted prevalence rate of outcome variables according to study groups and periods (%). ^a^<65 includes aged 50–64 years; ^b^≥65 includes aged 65–80 years

The prevalence of each outcome among comparison groups during the study periods have been presented in Figure 2. There was an improvement in measures of access to dental care (oral examination, preventive care, periodontal treatment, and tooth extraction), which increased in both eligible and non-eligible groups regardless of the income level.

### Main results of DD and DDD analyses

Table 2 shows the results for the most recent period (2016–2018) for the differences-in-differences analysis. Contrary to the initial hypothesis, expanded dental insurance led to an increase in unmet needs (8.8 percentage points, 95% confidence interval [CI]: 4.7 to 13.0), and a decrease in oral examination (6.0 percentage points; 95% CI, −9.0 to −2.0). However, there were improvements in dental prosthetics outcomes (denture wearing and dental implants) in the eligible group (*P* < 0.01; Table 2).

**Table 2.** Main results of DD and DDD analyses (%)

	Double Difference (DD) Net change after expansion			Triple Difference (DDD) Net change after expansion		
	
Outcome variables	Baseline Mean % in aged 65 over^a^	Unadjusted % (95% CI)	Adjusted % (95% CI)^b^	Baseline Mean % in aged 65 over with low-income^a^	Unadjusted % (95% CI)	Adjusted % (95% CI)^b^
Unmet dental needs	28.8	10.0^***^ (6.0 to 14.0)	8.8^***^ (4.7 to 13.0)	33.4	−19.6^***^ (−33.0 to −6.6)	−21.6^***^ (−35.0 to −8.5)
**Access to dental care**						
Oral examination	13.6	−8.1^***^ (−12.0 to −5.0)	−6.0^***^ (−9.0 to −2.0)	11.1	−7.6^***^ (−20.0 to −4.0)	−2.9 (−15.0 to 9.0)
Preventive care	3.3	−9.4^**^ (−17.0 to −2.0)	−9.8^**^ (−17.5 to −2.2)	2.8	−21.5 (−44.0 to 1.0)	−18.4 (−41.0 to 4.0)
Periodontal treatment	7.8	4.9 (−4.0 to 14.0)	1.1 (−8.2 to 10.4)	5.6	−14.6 (−41.0 to 11.0)	−16.7 (−43.0 to 9.5)
Extraction of teeth	10.0	6.9 (−2.0 to 15.0)	5.3 (−3.0 to 14.0)	11.1	−1.8 (−26.0 to 22.0)	−3.1 (−27.0 to 21.0)
**Clinical oral health outcomes**						
Periodontitis (CPITN)	42.3	1.1 (−3.0 to 5.0)	−0.3 (−5.0 to 4.0)	44.2	−1.2 (−16.0 to 13.0)	−2.0 (−17.0 to 13.0)
Number of remaining teeth	16.9	−0.2 (−0.9 to 0.4)	0.4 (−0.2 to 1.1)	15.9	−4.73^***^ (−7.0 to −2.4)	−3.8^***^ (−6.1 to −1.5)
Edentulous	9.3	0.6 (−1.0 to 2.0)	0.1 (−2.0 to 2.0)	11.1	4.8 (−1.0 to 11.0)	2.6 (−3.0 to 9.0)
Denture wearing	79.9	6.6^**^ (2.8 to 10.3)	4.0^***^ (0.2 to 7.9)	82.5	10.9 (−1.3 to 23.2)	9.1 (−3.4 to 21.6)
Denture needs	26.0	−1.6 (−4.8 to 1.7)	−4.4^***^ (−7.5 to −1.2)	26.7	−2.5 (−13.0 to 8.0)	−2.3 (−12.6 to 8.0)
Dental implants	11.4	4.0^**^ (0.9 to 7.1)	5.0^***^ (2.1 to 7.9)	8.8	−2.7 (−13.6 to 8.0)	−2.3 (−12.9 to 8.2)
**Perceived oral health outcomes related quality of life**						
Self-reported poor oral health	55.9	3.1 (−1.0 to 7.0)	1.5 (0.0 to 6.0)	58.7	1.6 (−12.0 to 16.0)	−1.3 (−15.0 to 13.0)

CI, confidence interval; CPITN, Community Periodontal Index of Treatment Needs.

Inference denotes: ^***^P < 0.01; ^**^P < 0.05; ^*^P < 0.1.

Means, DD, and DDD estimated for the most recent period (2016–2018) by linear regression.

^a^Baseline denotes 2010 year.

^b^Adjusted for sex, income and education in DD model, while sex and education in DDD model.

Although the 95% CI includes the null value in the DD model, the eligible group was more likely to access dental care (periodontal treatment and tooth extraction) than the non-eligible group after implementation of the policy. In addition, there was an improvement in the clinical oral health outcomes, except edentulism. The prevalence of periodontitis decreased by 0.3 percentage points (95% CI: −5.0 to 4.0) and the DD model showed an increase by 0.4 teeth in the number of teeth (95% CI: −0.2 to 1.1; Table 2).

Results from applying the DDD model showed that the insurance expansion led to a lower increase in unmet dental needs in the eligible low-income group, by 21.6 percentage points (95% CI: −35.0 to −8.5), compared to the high-income group. In contrast, there was a lower number of remaining teeth, by 3.8 (95% CI, −6.1 to −1.5) teeth in the low-income eligible group, compared to the high-income eligible group (*P* < 0.01; Table 2).

In the low-income eligible group, although the 95% CI includes the null value, expanded dental insurance was associated with a larger increase (by 9.1 percentage points) in the prevalence of denture wearing (95% CI, −3.4 to 21.6), compared to that seen in the high-income group. However, the expansion was likely to be associated with a smaller decline in the need for denture treatment (−2.3 percentage points; 95% CI: −12.6 to 8.0) and self-reported poor oral health (−1.3 percentage points; 95% CI: −15.0 to 13.0) among low-income adults compared to the high-income adults (Table 2).

### Robustness checks for the DD and DDD models

We conducted an empirical study using quasi-experimental analysis to establish an identification strategy that uses differences in oral health among groups before expansion of coverage to predict differences in oral health outcomes that would otherwise be expected in the absence of dental insurance expansion.^29^ However, the treatment and control groups might have been exposed to different influences at individual, community, and national level, which cannot be measured. Thus, we assumed that other predictors of oral health outcomes do not change among the non-eligible groups and differences between observed and expected oral health outcomes can largely be attributed to the effects of dental insurance policy expansion.

Results of the sensitivity analysis are shown in Table 3. The placebo period demonstrated a decrease in unmet dental needs in the DD (5.8 percentage points, 95% CI: −12 to 0.0) and the DDD models (6.0 percentage points, 95% CI: −23 to 13.0; *P* > 0.05).

**Table 3.** Sensitivity test for the DD and DDD models (%)

	Double Difference (DD) Net change after expansion			Triple Difference (DDD) Net change after expansion		
	
Outcome variables	Baseline Mean % in aged 65 over^a^	Unadjusted % (95% CI)	Adjusted % (95% CI)^b^	Baseline Mean % in aged 65 over with low-income^a^	Unadjusted % (95% CI)	Adjusted % (95% CI)^b^
**Prior period test** ^c^						
Unmet dental needs	32.9	−6.2 (−12.0 to 0.0)	−5.8 (−12 to 0.0)	41.2	−5.3 (−24.0 to 13.0)	−6.0 (−25.0 to 13.0)
**Alternative control test** ^d^						
Unmet dental needs	28.8	13.1^***^ (0.9 to 1.7)	12.3^***^ (8.3 to 16.4)	33.4	−1.1 (−12.7 to 10)	−3.5 (−18.4 to 11.4)
**Placebo outcome test** ^e^						
Perceived obesity	45.2	−0.90 (−5.0 to 3.0)	−0.50 (−5.0 to 4.0)	44.9	6.3 (−8.0 to 20.0)	6.5 (−7.0 to 21.0)

CI, confidence interval.

Inference denotes: ^***^P < 0.01; ^**^P < 0.05; ^*^P < 0.1.

Means, DD, and DDD estimated by linear regression.

^a^Baseline denotes 2010 year as a before the policy, except for the prior period test.

^b^Adjusted denotes sex, income and education in DD model, while sex and education in DDD model.

^c^The prior period was defined as year 2007, with year 2010 as the policy implementation year.

^d^Alternative control replaced aged 34–49 year instead of aged 50–64 year.

^e^Placebo outcome considered % of perceived obesity instead of the unmet dental needs.

On assessing the alternative control group, the results were found to robust and similar to our main analysis. The unmet dental needs increased by 12.3 percentage points (95% CI, 8.3–16.4) in the DD model (*P* < 0.01). For the placebo outcome test, there was a 95% confidence interval includes the zero for the prevalence of perceived obesity in either the DD or DDD model.

## DISCUSSION

Dental insurance expansion in Korea was associated with an overall increase in unmet dental needs among elderly individuals. However, the expansion was associated with a smaller increase in unmet dental needs among low-income older adults compared to high-income adults. To identify the causal effects of expanded dental insurance benefits for elderly individuals, we hypothesized that there would be no difference in unmet dental needs between the control and treatment groups before and after the introduction of the dental benefits policy in 2012. Additionally, we hypothesized that there would be no difference between the low- and high-income eligible groups before and after the introduction of the policy. Both the hypotheses for unmet dental needs were rejected using the DD and DDD quasi-experimental design.

However, contrary to our expectation, we found an increase in the unmet dental needs in the eligible group. This finding seemed inconsistent with the improved prevalence of denture wearing and dental implants (Table 2). However, this finding could be explained by two important factors—the demand and supply of dental services. The rise in expectations for receiving dental insurance benefits could have increased the reporting of dental needs among people. Older individuals may also have perceived increased unmet needs due to the persistence of out-of-pocket expenses that could not be covered by the insurance expansion. Further, provider-induced demand after the insurance expansion could be partially responsible for the increase in unmet dental needs.^35^^,^^36^ The expansion of health insurance has been shown to increase total medical expenses through supply-induced demand.^37^ Therefore, the increase in perceived unmet needs could have resulted from new diagnoses and treatment recommendations by dentists after an increase in patient visits to the clinic.^38^^,^^39^

Eligible individuals demonstrated an improvement in terms of three oral health outcomes in relation to dental prosthetics (denture wearing, denture needs, and dental implants) after insurance expansion. Expansion was also associated with a reduction in denture needs and an increase in the prevalence of denture-wearing and dental implants.

At the same time, two indicators of oral health moved in the “wrong” direction, including an increase in unmet dental needs concurrent with a decline in the number of remaining teeth. The DDD model revealed results favoring low-income individuals, with dental insurance benefits for elderly individuals increasing less in terms of unmet dental needs among the low-income eligible group, compared to the high-income group. This finding was consistent with that reported in previous studies examining expanded insurance benefits coverage, which partially improved income-related inequalities in tertiary care in South Korea.^40^ Improvements in perceived oral health outcomes might relate to the lower increase in unmet dental needs among low-income elderly individuals (Figure 2 and Table 2).^20^ Another plausible explanation could be increased tooth extractions due to an increased number of dental visits, which in turn lowered the level of dental needs.^22^^,^^41^ This phenomenon might be dubbed as a ‘paradox of missing teeth’. The need for root extractions prior to most prosthetic treatments could also explain the increase in the number of missing teeth. Evidence suggests that many individuals from less advantaged populations undergo tooth extractions, although alternative treatments are feasible in some cases.^42^ The resultant fewer number of teeth has largely been described as a cumulative consequence of dental disease over the lifetime among older individuals.^5^^,^^41^ Pre-existing endogenous factors also contribute to an increase in the number of missing teeth in low-income older adults. Thus, paradoxically, more missing teeth might be related to the decline of unmet dental needs among these older adults. Further evaluation of the policy with a long-term frame is needed to probe if it is transient or persistent for these untoward outcomes.

The findings of the present study point to the causal effect of expanded dental insurance on specific dental outcomes. Cost reduction for prosthetic treatments has a positive impact on denture wearing and dental implant treatment. Contrastingly, the reduction in oral examinations may be due to increase in the number of dental visits for other dental procedures (such as periodontal treatment, and teeth extraction; Figure 2 and Table 2).

Multiple sensitivity analyses were performed to ensure the robustness of the DD and DDD models. The validity of the study period was also verified to ensure that there had been no impact on unmet dental needs in the placebo period, as well as four indicators (oral examination, number of remaining teeth, edentulous, denture-wearing, and self-reported poor oral health) (eTable 2). Further, it was ensured that the outcome variables were valid for evaluating the impact of the expansion policy by considering prevalence of perceived obesity as a placebo outcome.

The use of narrower age-bands (60–64 vs 65–69 years, eTable 3) produced DD estimates that differed in magnitude, direction, and statistical significance, compared to our main findings (Table 2). This could be the result of unobserved heterogeneity (ie, confounding) due to the broader age bands used in our main analysis to define the treatment and control groups (65–80 years vs 50–64 years). Hence, caution is warranted in interpreting the DD estimates from the main analysis; our results may not be robust, even though we attempted to control for sex, education, and income.

There is a trade-off in defining treatment groups based on narrower age bands. On the one hand, the narrower age group increases the internal validity of the DD analysis by minimizing confounding. On the other hand, the generalizability (external validity) of the results is potentially affected because of focusing on only a narrow sub-group of the eligible population. For example, the DD estimates using the broader age bands suggested a statistically significant increase in the use of dentures and implants in the treatment group (Table 2); however, the estimates were not statistically significant (even though they were in the same direction) in the DD analysis using the narrower age band to define treatment and control groups (eTable 3). This may be because the use of broader age bands introduced confounding. Or alternatively, it could be because the policy did not affect the uptake of prosthetic treatment in the narrow 65–69 years age group, but it did so for the broader age-group (65–80 years).

In contrast to our DD analysis, our DDD estimates were broadly consistent, regardless of whether we used the narrower age bands or the broader age bands. The only exception was whether the participants reported an oral examination.

We confirmed that the estimates for the most recent period (2016–2018) were similar to the combined post-policy years (2012, 2014, 2015, and 2016–2018) for our outcome variables, with the exception of denture wearing and self-reported poor oral health. For instance, the DD and DDD estimates for unmet dental needs were changed slightly, from 8.8 percentage points to 6.3 percentage points, and from −21.6 percentage points to −15.9 percentage points in the adjusted DD and DDD model, respectively (eTable 4 and eFigure 1).

The DD and DDD design has some limitations. Our analysis may have residual bias due to differences in unobserved covariates that were not measured in the KNHANES data. The 2016–2018 period was considered a single point due to few participants at different times during the study period. Nevertheless, a quasi-experimental design was employed to assess causal inferences through identification that temporal changes in the expanded dental insurance policy are exogenous to changes in dental care needs and oral health outcomes. Importantly, we also found that expansion of dental insurance for elderly individuals may have led to a pro-poor impact for unmet dental needs.

### Conclusion

In conclusion, although expanded dental insurance for elderly individuals increased perceived unmet dental needs overall, the policy led to a substantially smaller increase in unmet needs among individuals in the low-income subgroup. In addition, insurance expansion led to significantly increased uptake of prosthetic dental treatment.

## SUPPLEMENTARY MATERIAL

eTable 1. Distribution of the study population according to study group (%)

eTable 2. Robustness checks with the prior period (2007 to 2009) for the DD and DDD models (%)

eTable 3. Robustness checks with the alternative control (aged 60–64) and treatment (aged 65–69) group for the DD and DDD models (%)

eTable 4. Main results of DD and DDD analyses (%)

eFigure 1. Unadjusted prevalence rate of outcome variables according to study groups and the most recent periods (2016–2018) (%). ^a^<65 includes aged 50–64 years. ^b^≥65 includes aged 65–80 years.
