# Supplementary material for: Did Dental Insurance Expansion Improve Dental Care Needs Among Korean Adults? Difference in Difference Analysis
Source: J Epidemiol. 2023 Feb 5;33(2):101–8. doi: 10.2188/jea.JE20200596 (PMC9794449; doi:10.2188/jea.JE20200596)
Supplement: Supplementary file 1 [file je-33-101-s001.pdf]

eTable 1. Distribution of the study population according to study group (%)

	Control (<65 years)[a]				Treated (≥65 years)[b]			
	total	**Before**[c]	**After**[d]	*P*	total	**Before**	**After**	*P*
Number of participants	5,451	1,795	3,656		7,019	1,478	3,363	
**Age**, years, mean (SE)		56.7 (0.10)	57.0 (0.07)			72.1 (0.12)	73.0 (0.09)	
**Sex**								
Men	2,359	43.3	43.2	0.95	2,095	43.9	43.0	0.55
Women	3,092	56.7	56.8		2,746	56.1	57.0	
**Education**								
Elementary	1,269	35.1	19.3	<0.01	2,835	68.5	59.1	<0.01
Middle school	986	22.5	17.3		640	12.0	14.9	
High school	1,767	29.4	36.2		708	12.9	16.6	
College over	1,177	13.1	27.2		392	6.6	9.4	
**Income**								
Low[e]	772	16.4	13.2	<0.01	2,438	53.3	49.7	<0.01
Middle-low	1,373	26.4	24.9		1,224	22.9	26.7	
Middle-high	1,484	26.7	27.8		687	12.9	14.9	
High[f]	1,784	30.5	34.1		451	11.0	8.7	

SE, standard error.
Unweighted number of the sample and weighted %.
[a]Control group (<65) included individuals aged 50–64 years.
[b]Treatment group (≥65) included individuals aged 65–80 years.

[c]Before the policy (2010 year).
[d]After the policy (2016–2018 year).
[e]Treatment group for triple difference (DDD) analysis.
[f]Control group for DDD analysis.

eTable 2. Robustness checks with the prior period (2007 to 2009) for the DD and DDD models (%)

	Double Difference (DD) Net change after expansion			Triple Difference (DDD) Net change after expansion		
**Outcome variables**	**Baseline Mean % in aged 65 over [a]**	**Unadjusted % (95% CI)**	**Adjusted % (95% CI) [b]**	**Baseline Mean % in aged 65 over with low-income [a]**	**Unadjusted % (95% CI)**	**Adjusted % (95% CI) [b]**
**Access to dental care**						
Oral examination	28.6	0.03 (-0.03 to 0.08)	0.03 (-0.03 to 0.08)	22.9	0.14 (-0.07 to 0.34)	0.13 (-0.07 to 0.33)
**Clinical oral health outcomes**						
Number of remaining teeth	16.0	-0.14 (-1.12 to 0.84)	-0.21 (-1.22 to 0.08)	15.2	-0.86 (-4.34 to 2.62)	-4.97 (-5.49 to 1.56)
Edentulous	11.4	0.001 (-0.03 to 0.03)	0.004 (-0.03 to 0.03)	12.2	0.04 (-0.06 to 0.13)	0.04 (-0.06 to 0.14)
Denture wearing	79.7	0.06 (-0.01 to 0.11)	0.03 (-0.02 to 0.09)	78.1	0.15 (-0.02 to 0.32)	0.14 (-0.04 to 0.33)
**Perceived oral health outcomes related quality of life**						
Self-reported poor oral health	54.4	0.002 (-0.06 to 0.06)	-0.01 (-0.07 to 0.06)	57.7	-0.06 (-0.28 to 0.15)	-0.06 (-0.27 to 0.16)

CI, confidence interval.
Inference denotes: *** P<0.01; ** P<0.05; * P<0.1.
Means, DD, and DDD estimated by linear regression.
[a]Baseline denotes 2007 year.
[b]Adjusted for sex, income and education in DD model, while sex and education in DDD model.
[c]Prior period defined as year 2007 with year 2009 as the policy implementation year.

eTable 3. Robustness checks with the alternative control (aged 60-64) and treatment (aged 65-69) group for the DD and DDD models (%)

Outcome variables	Double Difference (DD) Net change after expansion			Triple Difference (DDD) Net change after expansion		
	Baseline Mean % in aged 65 over [a]	Unadjusted % (95% CI)	Adjusted % (95% CI) [b]	Baseline Mean % in aged 65 over with low-income [a]	Unadjusted % (95% CI)	Adjusted % (95% CI) [b]
Unmet dental needs	32.3	-1.7 (-8.8 to 5.5)	-1.8 (-9.0 to 5.4)	41.0	-24.5** (-45.6 to -3.3)	-25.5** (-46.8 to 4.2)
**Access to dental care**						
Oral examination	16.4	-1.9 (-8.2 to 4.3)	-2.1 (-8.4 to 4.2)	12.6	20.0 (-0.9 to 40.9)	21.8** (0.8 to 42.7)
Preventive care	0.0	9.2 (-2.2 to 20.5)	8.5 (-3.6 to 20.6)	0.0	-14.1 (-51.5 to 23.2)	-8.6 (-51.5 to 34.4)
Periodontal treatment	7.7	-1.2 (-13.7 to 11.4)	-4.7 (-17.7 to 8.4)	7.7	-25.1 (-65.0 to 14.9)	-17.0 (-57.6 to 23.6)
Extraction of teeth	7.7	2.5 (-9.9 to 14.9)	-3.5 (-17.0 to 10)	7.7	-0.75 (-46.0 to 31.1)	0.6 (-40.3 to 41.6)
**Clinical oral health outcomes**						
Periodontitis (CPITN)	42.3	-3.1 (-10.6 to 4.4)	-3.7 (-11.3 to 3.9)	45.3	16.7 (-7.1 to 40.5)	18.1 (-6.1 to 42.3)
Number of remaining teeth	19.8	0.2 (-0.9 to 1.3)	0.1 (-1.0 to 1.2)	19.4	-3.69** (-7.3 to 0.0)	-3.7** (-7.3 to 0.0)
Edentulous	3.6	0.1 (-2.3 to 2.6)	-0.04 (-2.6 to 2.5)	4.2	-0.8 (-7.8 to 6.2)	-0.6 (-8.0 to 6.7)
Denture wearing	76.1	2.6	1.4	76.4	12.1	11.4

		(-4.0 to 9.2)	(-5.4 to 8.3)		(-8.7 to 32.9)	(-10 to 32.8)
Denture needs	23.7	-0.02<br>(-7.6 to 3.6)	-4.2<br>(-9.7 to 1.2)	26.1	-1.0<br>(-18.1 to 16.0)	4.9<br>(-11.5 to 21.3)
Dental implants	11.9	4.3<br>(-1.5 to 10)	2.7<br>(-2.8 to 8.3)	10.4	9.9<br>(-9.0 to 28.8)	8.1<br>(-10.3 to 26.6)
**Perceived oral health outcomes related quality of life**						
Self-reported poor oral health	54.4	-2.9<br>(-10.4 to 4.5)	-2.8<br>(-10.3 to 4.7)	59.3	9.3<br>(-14.2 to 32.8)	10.8<br>(-12.7 to 34.3)

CI, confidence interval; CPITN, Community Periodontal Index of Treatment Needs.
Inference denotes: *** P<0.01; ** P<0.05; * P<0.1.
Means, DD, and DDD estimated by linear regression.
[a]Baseline denotes 2010 year as a before the policy.
[b]Adjusted denotes sex, income and education in DD model, while sex and education in DDD model.

eTable 4. Main results of DD and DDD analyses (%)

**2012–2018**	**Double Difference (DD) Net change after expansion**			**Triple Difference (DDD) Net change after expansion**		
**Outcome variables**	**Baseline Mean % in aged 65 over [a]**	**Unadjusted % (95% CI)**	**Adjusted % (95% CI) [b]**	**Baseline Mean % in aged 65 over with low-income [a]**	**Unadjusted % (95% CI)**	**Adjusted % (95% CI) [b]**
Unmet dental needs	28.8	6.9*** (3.3 to 10.6)	6.3*** (2.6 to 10.0)	33.4	-14.4*** (-25.6 to -3.1)	-15.9*** (-27.3 to -4.5)
**Access to dental care**						
Oral examination	13.6	-6.8*** (-9.8 to -3.8)	-5.4*** (-8.5 to -2.4)	11.1	-5.7 (-16.1 to -4.6)	-2.6 (-13.1 to 7.8)
Preventive care	3.3	4.5 (-7.2 to 16.2)	2.4 (-10.0 to 14.8)	2.8	-1.2 (-38.3 to 35.8)	-3.4 (-42.2 to 35.3)
Periodontal treatment	7.8	13.6** (0.8 to 26.3)	8.6 (-5.0 to 22.3)	5.6	-3.4 (-43.0 to 36.2)	-6.6 (-47.5 to 34.3)
Extraction of teeth	10.0	14.6** (1.8 to 27.4)	7.4 (-6.5 to 21.4)	11.1	10.1 (-28.7 to 49.0)	6.3 (-34.0 to 46.6)
**Clinical oral health outcomes**						
Periodontitis (CPITN)	42.3	-0.1 (-4.0 to 3.9)	-0.5 (-4.6 to 3.5)	44.2	-2.8 (-15.9 to 10.3)	-2.1 (-15.3 to 11.1)
Number of remaining teeth	16.9	0.2 (-0.4 to 0.8)	0.6 (-0.02 to 1.2)	15.9	-4.1*** (-6.1 to -2.0)	-3.1** (-5.2 to -1.1)
Edentulous	9.3	-0.1 (-1.9 to 1.6)	-0.7 (-2.5 to 1.1)	11.1	3.9 (-1.6 to 9.5)	1.8 (-3.7 to 7.3)
Denture wearing	79.9	2.2 (-1.2 to 5.5)	1.5 (-2.1 to 5.0)	82.5	3.1 (-7.5 to 13.7)	3.4 (-7.6 to 14.4)
Denture needs	26.0	-1.5	-3.4**	26.7	-4.1	-1.9

		(-4.6 to 1.6)	(-6.5 to -0.4)		(-14.2 to 6.0)	(-11.6 to 7.9)
Dental implants	11.4	2.2<br>(-0.5 to 4.9)	2.8** (0.3 to 5.3)	8.8	-8.5<br>(-17.8 to 0.8)	-6.2<br>(-15.1 to 2.8)
**Perceived oral health outcomes related quality of life**						
Self-reported poor oral health	55.9	2.2<br>(-0.5 to 4.9)	2.8** (0.3 to 5.3)	58.7	-8.5<br>(-17.8 to 0.8)	-6.2<br>(-15.1 to 2.8)

CI, confidence interval; CPITN, Community Periodontal Index of Treatment Needs.

Inference denotes: *** P<0.01; ** P<0.05; * P<0.1.

Means, DD, and DDD estimated for the combined post-policy years (2012, 2014, 2015, and 2016–2018) by linear regression.

[a]Baseline denotes 2010 year.

[b]Adjusted for sex, income and education in DD model, while sex and education in DDD model.

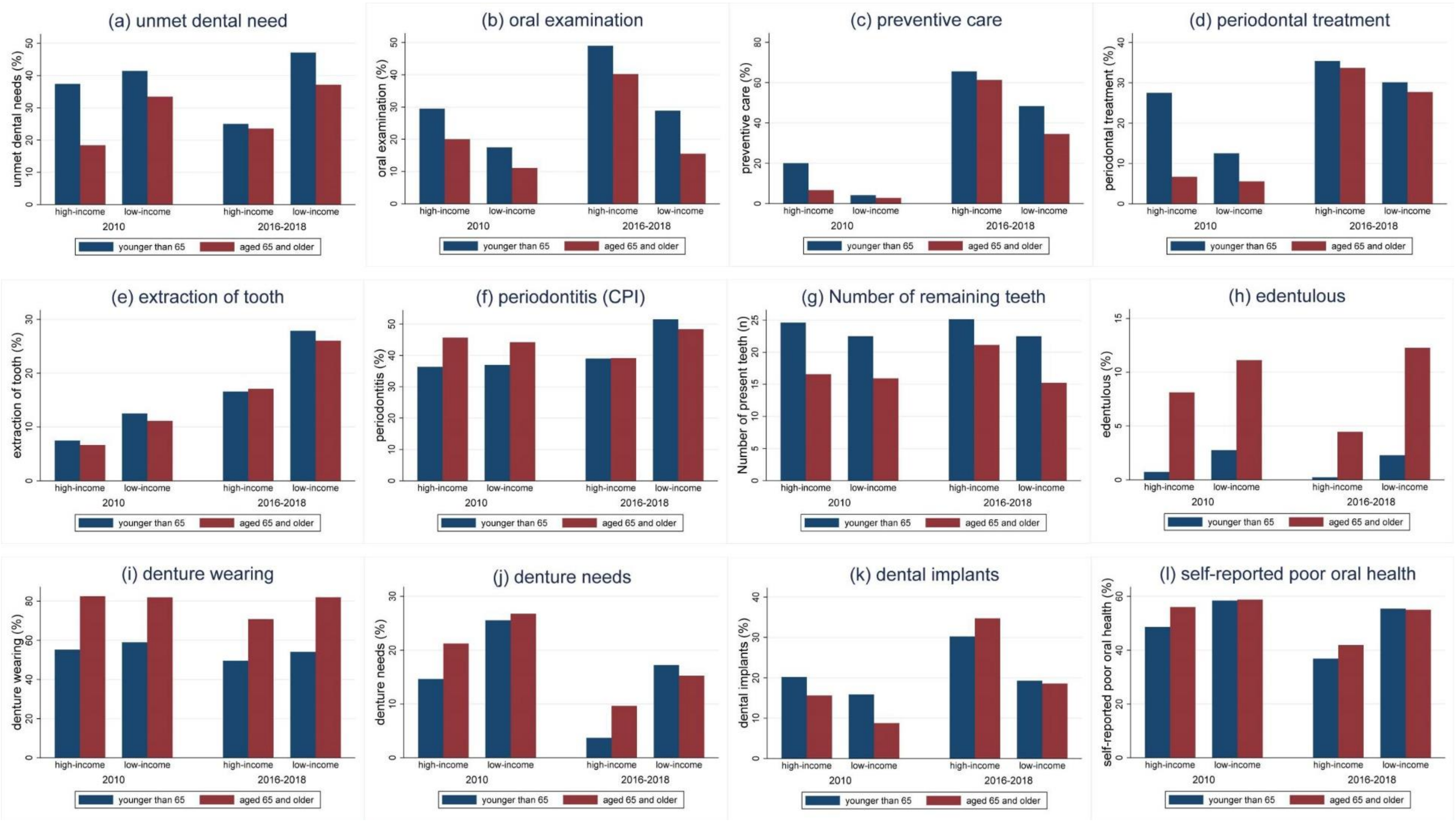

eFigure 1. Unadjusted prevalence rate of outcome variables according to study groups and the most recent periods (2016–2018) (%). [a] <65 includes aged 50–64 years. [b] ≥65 includes aged 65–80 years.
